# Retraction

**DOI:** 10.1002/hsr2.1731

**Published:** 2023-11-20

Retraction: [*Prevalence of active trachoma infection and associated factors post‐war resettled population in raya kobo districts, North East Ethiopia: A community‐based cross‐sectional study in 2022*. F. Kebede, M. Jamal. *Health Science Reports*. 2023. 10.1002/hsr2.1486.]

The above article, published online on 06 August 2023 in Wiley Online Library (wileyonlinelibrary.com), has been retracted by agreement between the journal's Editor‐in‐Chief, Dr Charles Young, and John Wiley & Sons, Inc. The retraction has been agreed due to major overlap with a previously published article from the same group of authors.

